# A Multicenter Study of the Validity and Reliability of Responses to Hand Cold Challenge as Measured by Laser Speckle Contrast Imaging and Thermography: Outcome Measures for Systemic Sclerosis–Related Raynaud's Phenomenon

**DOI:** 10.1002/art.40457

**Published:** 2018-04-23

**Authors:** Jack D. Wilkinson, Sarah A. Leggett, Elizabeth J. Marjanovic, Tonia L. Moore, John Allen, Marina E. Anderson, Jason Britton, Maya H. Buch, Francesco Del Galdo, Christopher P. Denton, Graham Dinsdale, Bridgett Griffiths, Frances Hall, Kevin Howell, Audrey MacDonald, Neil J. McHugh, Joanne B. Manning, John D. Pauling, Christopher Roberts, Jacqueline A. Shipley, Ariane L. Herrick, Andrea K. Murray

**Affiliations:** ^1^ University of Manchester Manchester Academic Health Science Centre Manchester UK; ^2^ University of Manchester, Manchester, UK, and Salford Royal Foundation, NHS Trust, Manchester Academic Health Science Centre Salford UK; ^3^ Freeman Hospital and Newcastle University Newcastle upon Tyne UK; ^4^ University of Liverpool Liverpool UK; ^5^ Leeds Teaching Hospitals NHS Trust Leeds UK; ^6^ NIHR Leeds Musculoskeletal Biomedical Research Unit and Leeds Institute of Rheumatic and Musculoskeletal Medicine Chapel Allerton Hospital Leeds UK; ^7^ University College London Medical School London UK; ^8^ Freeman Hospital Newcastle upon Tyne Hospitals NHS Foundation Trust Newcastle upon Tyne UK; ^9^ Cambridge University Hospitals NHS Foundation Trust Cambridge UK; ^10^ Royal National Hospital for Rheumatic Diseases Royal United Hospitals NHS Foundation Trust Bath UK; ^11^ Royal National Hospital for Rheumatic Diseases Royal United Hospitals NHS Foundation Trust and University of Bath Bath UK; ^12^ University of Manchester and NIHR Manchester Musculoskeletal Biomedical Research Centre Central Manchester NHS Foundation Trust Manchester Academic Health Science Centre Manchester UK; ^13^ Salford Royal Foundation, NHS Trust, Manchester Academic Health Science Centre Salford UK

## Abstract

**Objective:**

Reliable and objective outcome measures to facilitate clinical trials of novel treatments for systemic sclerosis (SSc)–related Raynaud's phenomenon (RP) are badly needed. Laser speckle contrast imaging (LSCI) and thermography are noninvasive measures of perfusion that have shown excellent potential. This multicenter study was undertaken to determine the reliability and validity of a hand cold challenge protocol using LSCI, standard thermography, and low‐cost cell phone/mobile phone thermography (henceforth referred to as mobile thermography) in patients with SSc‐related RP.

**Methods:**

Patients with RP secondary to SSc were recruited from 6 UK tertiary care centers. The patients underwent cold challenge on 2 consecutive days. Changes in cutaneous blood flow/skin temperature at each visit were imaged simultaneously using LSCI, standard thermography, and mobile thermography. Measurements included area under the curve (AUC) for reperfusion/rewarming and maximum blood flow rate/skin temperature after rewarming (MAX). Test–retest reliability was assessed using intraclass correlation coefficients (ICCs). Estimated latent correlations (estimated from multilevel models, taking values between −1 and 1; denoted as rho values) were used to assess the convergent validity of LSCI and thermography.

**Results:**

In total, 159 patients (77% with limited cutaneous SSc) were recruited (84% female, median age 63.3 years). LSCI and standard thermography both had substantial reliability, with ICCs for the reperfusion/rewarming AUC of 0.67 (95% confidence interval [95% CI] 0.54, 0.76) and 0.68 (95% CI 0.58, 0.80), respectively, and ICCs for the MAX of 0.64 (95% CI 0.52, 0.75) and 0.72 (95% CI 0.64, 0.81), respectively. Very high latent correlations were present for the AUCs of LSCI and thermography (ρ = 0.94; 95% CI 0.87, 1.00) and for the AUCs of standard and mobile thermography (ρ = 0.98; 95% CI 0.94, 1.00).

**Conclusion:**

This is the first multicenter study to examine the reliability and validity of cold challenge using LSCI and thermography in patients with SSc‐related RP. LSCI and thermography both demonstrated good potential as outcome measures. LSCI, standard thermography, and mobile thermography had very high convergent validity.

Systemic sclerosis (SSc)–related digital vasculopathy is painful and disabling, and has significant impact on quality of life. Raynaud's phenomenon (RP) occurs in most patients with SSc (96%) and is consistently the highest ranked symptom of SSc in terms of frequency and impact on daily function 1, 2. In patients with SSc, RP often progresses to severe digital vasculopathy, with up to 50% of patients developing painful digital ulceration 3, 4, 5, 6, 7, 8, 9, 10, 11.

Treatments are far from ideal, and Cochrane reviews (http://www.cochranelibrary.com) as well as other reviews have highlighted the lack of evidence base for the treatment of both primary and SSc‐related RP 12, 13, 14, 15. One of the reasons for this shortcoming is the lack of reliable outcome measures, which are necessary to deliver successful clinical trials. Technological advances in laboratory measurements of blood flow (laser speckle contrast imaging [LSCI] and thermography [skin temperature, a pseudo measure of perfusion]) hold promise as objective outcome measures 16, 17. The Outcome Measures in Rheumatology 6 (OMERACT 6) report, describing the current status of outcome measure development for clinical trials in SSc, concluded that whether imaging techniques made the transition from research pathophysiologic measurement techniques to outcome measures for RP was dependent on whether “data are published or available to show their validity” 18. The requirement for reliable outcome measures to facilitate highly powered clinical trials in SSc‐related RP is now especially pertinent due to ongoing novel drug developments 19, 20, 21, 22, 23. Whereas patient‐reported outcome measures such as the Raynaud's condition score (RCS; a measure of RP disease activity with a possible score range of 0–10, with higher scores indicating more active disease) 24 are well suited for later (i.e., phase III) studies, objective, noninvasive imaging techniques would provide confirmatory testing to inform stop–go decision‐making in earlier (i.e., phase II) studies.

Our main aim in the present study was to determine whether LSCI and thermography, performed subsequent to application of a cold challenge to the hands, are sufficiently reliable and valid to allow their use as outcome measures in multicenter clinical trials. Our primary objectives were to evaluate test–retest reliability and construct validity 25, which we defined as the ability of LSCI and thermography to measure important features of SSc‐related digital vasculopathy. Our secondary objectives were to assess the interobserver reliability, as well as feasibility, of the techniques. Just prior to commencement of our study, cell phone/mobile phone thermography (henceforth referred to as mobile thermography) came on the market as an imaging method, potentially offering a more cost‐effective and portable alternative to LSCI and “standard” thermography. Thus, an additional secondary objective was to assess the utility of mobile thermography in comparison to standard thermography.

## Patients and Methods

**Patients.** Six UK tertiary care centers that provide clinical care to patients with SSc took part in the study. Individuals responsible for imaging and analysis attended a central training session prior to the start of recruitment. At least one person from each center attended the training.

The study aimed to recruit 180 patients with SSc (for the inclusion and exclusion criteria used, including current digital ulceration, see Supplementary Table 1, available on the Arthritis & Rheumatology web site at http://onlinelibrary.wiley.com/doi/10.1002/art.40457/abstract). The study was approved by the Cambridgeshire and Hertfordshire National Research Ethics Service Committee (approval number 15/EE/0083), and all patients gave written consent to participate.

All patients were recruited between October 1, 2015 and February 28, 2016, to minimize interindividual variation related to season. Each visit took ~1 hour.

**Figure 1:**
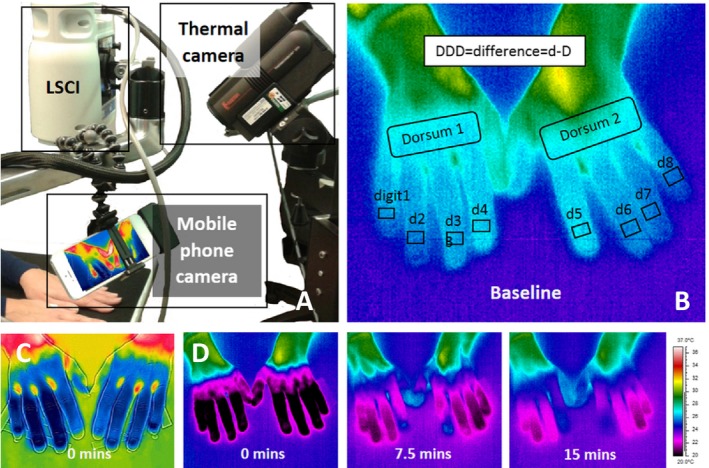
**A,** Photograph of the imaging equipment set up to allow simultaneous imaging, showing laser speckle contrast imaging (LSCI), standard thermography, and mobile thermography. **B,** Baseline image of the hands obtained with standard thermography, showing distal dorsal difference (DDD) regions of interest, with fingers being cooler than dorsum. **C,** An example of hands imaged by mobile thermography at 0 minutes post–cold challenge, with fingers being cooler than dorsum (scale unavailable for image due to the software used). **D,** An example of hands undergoing rewarming (same subject as in **B**) imaged by the standard thermal camera at 0 minutes, 7.5 minutes, and 15 minutes after cooling. Scale on the right refers to the temperature range (20–37°C) shown in **B** and **D**.

**Figure 2:**
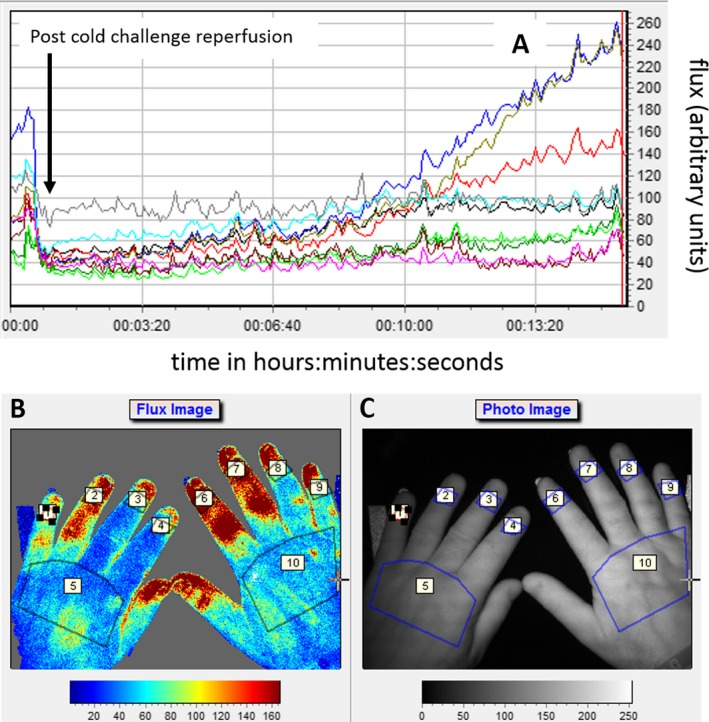
**A,** Laser speckle contrast imaging (LSCI) reperfusion graphs for 8 digits (regions of interest [ROIs] 1–4 and 6–9, as shown in **B**) and 2 dorsa (ROIs 5 and 10). Graphs show reperfusion post–cold challenge (i.e., flux, which was proportional to the product of the average speed of the blood cells and their concentration, expressed in arbitrary perfusion units) versus time. **B,** Example of a flux image (i.e., perfusion map) showing ROIs marked (as described in Figure 1). **C,** Photograph of the hands showing the ROIs assessed by LSCI.

**Imaging equipment.** An LSCI thermal camera (FLPI‐2; Moor Instruments) 16, 17 was leased to each center (Figures 1A and 2B). Five of the 6 centers used their own thermal cameras (referred to as “standard thermography”) (Figures 1A, B, and D) 26, and the sixth center leased a camera. A mobile phone/device–connectable thermography camera (FLIR One) (Figures 1A and C) and an Apple iPhone 5 were purchased for each center, along with all other cold challenge equipment, to minimize variation between centers. Furthermore, to minimize differences between centers, equipment at each site was set up according to strict guidelines for positioning to ensure images were obtained in as similar a manner as possible (in terms of angles and distances), and a calibration protocol was applied to the equipment at the start and end of the study (carried out by a single person from the central site [EJM]). LSCI settings were adjusted for distance, frequency, duration, focus, intensity overlay, processing mode (high resolution), and color image acquisition. Thermal camera settings were adjusted for room temperature, distance to hands, and skin emissivity. Mobile thermography settings were limited but the “matte” emissivity setting was chosen.

**Cold challenge.** Patients were requested to wear light clothing and refrain from vigorous exercise, caffeine, and alcohol for 4 hours prior to the assessment. Upon arrival, patients were seated comfortably for 20 minutes and acclimatized, and clinical research forms were completed. Immediately prior to the cold challenge, a baseline image of both hands (dorsal aspect) was obtained with LSCI and both thermal cameras. As required for LSCI imaging, all images were acquired in low‐lit rooms.

The patient's hands were placed on a black, thermally insulated surface (1 meter away from the thermal cameras and 70 cm [±5 cm] from the LSCI). Small sticky dots were used to mark the location of each finger at baseline. Both hands were placed in nitrile gloves and immersed to the metacarpophalangeal joints for 1 minute into cooled water (temperature of 15°C [±1°C], measured by calibrated thermometer) in 2 standard containers, one on either side of the patient. After the cold challenge, the gloves were removed and the hands were returned to their original position on the insulating surface, secured by double‐sided sticky tape to avoid movement between images.

Reperfusion/rewarming after application of the cold challenge was imaged simultaneously by LSCI at 15 frames per minute, and thermography at 4 frames per minute, for 15 minutes (i.e., contemporaneous measurement for 15 minutes postcooling). Mobile thermography did not allow for continuous video images to be obtained, and thus single images from which data could be extracted were acquired at set time points: baseline, 0 minutes after cold challenge, and 15 minutes after cold challenge. At the end of the 15 minutes, 1 extra image was obtained for LSCI and standard thermography, to allow the reperfusion/rewarming gradient from the last data point to be calculated; thus, a total of 225 images/scans were obtained for LSCI, 61 for thermography, and 3 for mobile thermography during the 15 minutes of measurement.

Analysis of the images was performed using Moor Instruments Laser Perfusion Imager software (version 4.0) for LSCI, and Research IR Max (version 4.2; FLIR) for standard and mobile thermography. Patients completed the RCS (possible score range 0–10) at each visit (a measure referred to as “RCS on the day”), measuring the severity and impact of their RP for that day 24.

**Figure 3:**
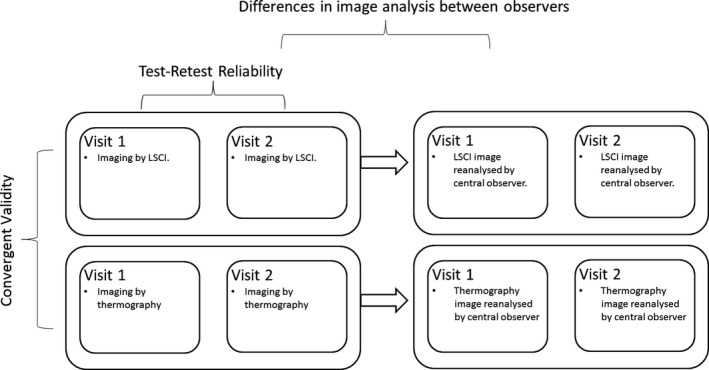
Study design. The images obtained were assessed for convergent validity, test–retest reliability, and interobserver differences. LSCI = laser speckle contrast imaging.

The cold challenge was repeated 1 day later (on day 2), as close as possible to the same time of day in order to minimize variation due to circadian rhythms 27. The repetition over 2 consecutive days (i.e., ~24 hours) minimized any variations within individuals over time (e.g., menstrual cycle effects) and seasonal variation in weather 28. Five centers had 1 observer, while 1 center had 2 observers. Each examiner re‐examined the same subject on days 1 and 2; for example, at the central site, 1 observer imaged 60 patients, twice, on consecutive days. Figure 3 shows the study design.

**Figure 4:**
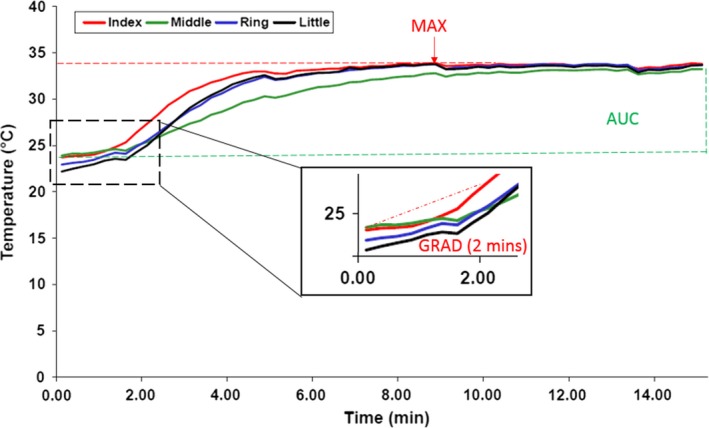
Example of a reperfusion/rewarming area under the curve (AUC), maximum blood flow rate/skin temperature after rewarming (MAX), and gradient of reperfusion/rewarming in the first 2 minutes post–cold challenge (GRAD) for 1 hand, measured with standard thermography. The data for the index, middle, ring, and little finger are shown as 4 solid lines, one for each finger (regions of interest were confined to the 8 distal phalanges, as indicated in Figure 2B). Color figure can be viewed in the online issue, which is available at http://onlinelibrary.wiley.com/doi/10.1002/art.40457/abstract.

**Image analysis for summary measures of response.** Image analysis was carried out locally by an internal, nonblinded observer at each center. These were the same observers who had obtained the images. Regions of interest (ROIs) (Figures 1B and 2B and C) were highlighted in the baseline (pre–cold challenge) image and in sequential images for 15 minutes postcooling. The distal dorsal difference (DDD) (defined as the difference in measurements between the dorsum and the finger, with DDD_L_, DDD_T_, and DDD_M_ representing the values based on LSCI, standard thermography, and mobile thermography, respectively) 29, 30 was calculated for each finger at baseline. In the sequential images, the ROIs were confined to the 8 distal phalanges. The area under the curve (AUC) for reperfusion/rewarming in each finger (with AUC_L_, AUC_T_, and AUC_M_ representing the values based on LSCI, standard thermography, and mobile thermography, respectively) was calculated manually, not by automation (Figure 4) (standard thermography), from 61 postchallenge images. In addition, the maximum blood flow rate/skin temperature after rewarming (MAX; with MAX_L_, MAX_T_, and MAX_M_ representing the values based on LSCI, standard thermography, and mobile thermography, respectively) and the gradient of reperfusion/rewarming in the first 2 minutes post–cold challenge (GRAD; with GRAD_L_, GRAD_T_, and GRAD_M_ representing the values based on LSCI, standard thermography, and mobile thermography, respectively) were determined. Data were averaged for all fingers, as was done in previous studies 16. For mobile thermography, the DDD was obtained from the first of 3 images, and the AUC was approximated by averaging the data over the latter 2 images. Analysis took <1 hour per participant, per visit.

Saved images and ROI local analysis data were also analyzed by the blinded central observer (TLM). Mobile thermography image analysis was carried out only at the central site.

**Assessment of feasibility.** The feasibility of each technique was assessed at the end of the study based on the individual opinion of the clinical scientist or technician. Feasibility was measured as the ease of use and the ease of analysis (score scale for each 0–10, where 0 = difficult, 10 = easy), and preference of LSCI over thermography (standard or mobile thermography) for acquiring and analyzing images.

**Measurement of room temperature.** A prerequisite of the cold challenge, and thus a criterion for center participation, was a temperature‐controlled room at each center. All measurements were obtained in a temperature‐controlled room (aimed at a room temperature of 23°C [± 2°C]. Room temperatures were recorded with data monitors (TinyTag; Gemini Data Loggers) to assess the impact of temperature on measurements, with an interest in examining whether reliability could be improved by achieving greater temperature control.

**Identification of edge effects from LSCI.** It became apparent when the study began that the blood flow appeared to be lower at the edges of the LSCI images than in the center. This implied that the distribution of the laser light across the hands was not equal, with less light incident toward the edges than at the center of the image. If true, then the consequence of this would be an artificially lower value for perfusion in the little fingers (edge of image) (Figure 2B) compared to index fingers (center of image) in the LSCI images. Thus, this was investigated further, as detailed below.

**Statistical analysis.** *Sample size calculation*. Analyses of the data were performed using R version 3.2.3 31. Based on observations in a previous thermography study 16, a sample size of 180 patients would allow us to estimate the test–retest reliability to within 0.05. A full discussion of the sample size calculation and other aspects of the statistical analyses (extended statistical analysis) are available from the corresponding author upon request.

*Determination of test–retest reliability*. To determine the test–retest reliability of each technique, intraclass correlation coefficients (ICCs) were obtained using linear mixed effects models. Each summary measure was included as a dependent variable, with center included as a fixed effect.

*Determination of between‐observer reliability*. The data over both visits for each patient were averaged, and the resulting average values were compared between the central observer and the center‐specific observers by calculating the difference (with 95% confidence interval [95% CI]) in the paired mean values (details available from the corresponding author upon request). It is not possible to calculate a valid interobserver ICC from these data, since it would require at least some of the participants to have traveled to all sites for imaging and a large subset of images to be analyzed by all observers 32.

*Determination of validity of the techniques*. Convergent validity (one aspect of construct validity) was assessed using bivariate linear mixed models, which included fixed center terms and separate random patient intercepts for 1) LSCI and standard thermography, and 2) standard and mobile thermography. We estimated the latent correlation coefficients (if the techniques measured the same construct, the latent correlation would be a rho value of 1). For clarity, the statistical analysis protocol for this joint model is available from the corresponding author upon request.

A post hoc analysis was conducted in which the responses to the RCS corresponding to the study day were assessed for correlation with all measurements, using linear mixed models.

*Feasibility data*. Descriptive statistics were used to summarize the feasibility data.

*Mixed effects models accounting for room temperature*. The mean room temperature at each patient visit was added to the mixed effects models for each summary measure. ICCs were recalculated, and these values were compared to the previously calculated estimates.

*Analysis of edge effects*. Edge effects were investigated in a post hoc analysis by calculating the trend across fingers for LSCI measurements, and then comparing these measurements to those from thermography. Linear mixed models were used to assess any linear trends in the measurements from the index finger to the little finger. Fingers were numbered. Finger‐level summary measures of response were then regressed on finger number for both LSCI and thermography; this linear approximation was crude, but sufficient. Random intercept and slope terms were included to account for the fact that there was variation from patient to patient in these trends, not attributable to the imaging techniques. Measurements were standardized prior to analysis, thereby allowing for comparisons to be made between LSCI and thermography.

## Results

**Characteristics of the patients.** In total, 159 patients were recruited (60 from the central center, 16–20 from each of the other centers). Among the patients, 157 (99%) fulfilled the American College of Rheumatology/European League Against Rheumatism 2013 classification criteria for SSc 33. The median age of the patients was 63.3 years (interquartile range [IQR] 53.8–69.5 years) and 123 (77%) had limited cutaneous SSc 34. The median disease duration since first non‐Raynaud's symptom was 9.6 years (IQR 4.5–17.4 years). Of the 159 patients with SSc, 142 (89%) were receiving treatment with vasodilators (61 with calcium channel blockers, 27 with angiotensin‐converting enzyme inhibitors, 27 with angiotensin II receptor antagonist, 22 with phosphodiesterase 5 inhibitor, 4 with endothelin receptor antagonist, and 1 with nitrates), of whom 35 were receiving ≥1 vasodilator. Moreover, 4 patients (3%) had previously undergone finger surgical debridements, 5 (3%) had previously had amputations, and 30 (19%) had experienced ulcers in the preceding year.

**Test–retest reliability of the techniques.** There was at least moderate to substantial reliability in the DDD_L_, DDD_M_, and DDD_T_, the AUC_L,_ AUC_T_, and AUC_M_, and the MAX_L_ and MAX_T_. The GRAD_L_ and GRAD_T_ had fair to substantial test–retest reliability (Table 1). The strength of reliability was assessed according to previously defined score classifications (see ref. 35), as follows: ICC 0.00–0.20 = slight, ICC 0.21–0.40 = fair, ICC 0.41–0.60 = moderate, ICC 0.61–0.80 = substantial, and ICC 0.81–1.00 = almost perfect. However, these classifications are, to some extent, arbitrary and should be treated as a rough guide.

**Table 1:** Reliability and validity of LSCI and thermography (standard and mobile phone–based) in patients with systemic sclerosis–related Raynaud's phenomenon^a^

Summary measure	Test–retest reliability			Difference in reliability,	Validity	
	LSCI (n = 159)	Standard thermography (n = 159)	Mobile phone thermography (n = 141)^b^	LSCI versus standard thermography	LCSI and standard thermography	Standard and mobile phone thermography
Distal dorsal difference	0.67 (0.56, 0.77)	0.58 (0.43, 0.71)	0.61 (0.51, 0.73)	0.08 (−0.05, 0.25)	0.65 (0.50, 0.79)	0.90 (0.79, 0.97)
Reperfusion/rewarming AUC_log_	0.67 (0.54, 0.76)	0.68 (0.58, 0.80)	0.61 (0.51, 0.72)^c^	−0.01 (−0.17, 0.11)	0.94 (0.87, 1.00)	0.98 (0.94, 1.00)
MAX_log_	0.64 (0.52, 0.75)	0.72 (0.64, 0.81)	NA	−0.09 (−0.21, 0.03)	0.87 (0.77, 0.95)	NA
Gradient over first 2 minutes	0.46 (0.40, 0.69)	0.56 (0.40, 0.74)	NA	−0.09 (−0.24, 0.18)	0.52 (0.33, 0.70)	NA

a Data for the summary measures of distal dorsal difference, reperfusion/rewarming area under the curve (AUC_log_), maximum blood flow rate/skin temperature after rewarming (MAX_log_), and gradient of reperfusion/rewarming over the first 2 minutes have been averaged over 8 digits. Values are the intraclass correlation coefficients (ICCs) (with 95% confidence intervals [95% CIs]) for the test–retest reliability of laser speckle contrast imaging (LSCI), standard thermography, and mobile phone–based thermography, the difference (with 95% CIs) in ICC point estimates between LSCI and standard thermography, and the estimated validity, expressed as latent correlation coefficients (with 95% CIs), between LSCI and standard thermography and between standard and mobile phone thermography. NA = not applicable.

b For mobile phone thermography, 141 data sets were available (n = 18 missing due to technical fault at 2 of the centers).

c The AUC_log_ for mobile phone thermography was approximated from the mean values of 2 frames, post–cold challenge of the hand.

**Reliability between observers.** When the data from each visit, observer, and center, and additionally at the patient level, were analyzed for reliability (see Supplementary Table 2 and Supplementary Figure 1, available on the Arthritis & Rheumatology web site at http://onlinelibrary.wiley.com/doi/10.1002/art.40457/abstract), we considered that if the measures were perfectly reliable, the subplot for each center would look like 2 identical ladder plots (but it is not expected that the plots would be identical between centers). Our data suggest that there were systematic differences between the central observer and one of the centers (center 2, and possibly center 3 [details available from the corresponding author upon request]) in extracting data from LSCI images. For thermography, agreement between the central and local observer was generally high for all centers, albeit with a large discrepancy in the data from several patients at one of their visits (results available from the corresponding author upon request).

**Validity of the techniques.** The latent correlation between LSCI and standard thermography (i.e., evidence that LSCI and standard thermography measure the same construct, which, in this case, was blood flow returning to the finger after cold challenge) was as follows: for the DDD, ρ = 0.65 (95% CI 0.50, 0.79); for the reperfusion/rewarming AUC, ρ = 0.94 (95% CI 0.87, 1.00); and for the MAX, ρ = 0.87 (95% CI 0.77, 0.95). In contrast, for the GRAD, the latent correlation between LSCI and standard thermography was only 0.52 (95% CI 0.33, 0.70) (Table 1). High latent correlation is indicative of convergent validity. Therefore, a value of 0.7 could be considered high, such that both the MAX and the AUC displayed strong convergent validity.

Correlation between mobile thermography and standard thermography was also very high. The latent correlation was 0.98 (95% CI 0.94, 1.00) for the AUC, and 0.90 (95% CI 0.79, 0.97) for the DDD (Table 1). Latent correlation between LSCI and mobile thermography was 0.86 (95% CI 0.74, 0.97) for the AUC, and 0.49 (95% CI 0.29, 0.66) for the DDD.

With the exception of some weak evidence of a decreasing DDD as measured on thermography with increasing RCS score (mean ± SEM change in the DDD_T_ of −0.15 ± 0.07 for a 1‐point increase in the RCS), we found no evidence of correlation between the summary measures and the RCS.

**Feasibility.** Standard thermography was deemed to be more feasible than LSCI (see Discussion). The proportion of raters giving a score of ≥7 for ease of use was 50% for LSCI, 75% for standard thermography, and 38% for mobile thermography. Ease of analysis was rated as ≥7 by 25% of raters for LSCI and by 50% of raters for standard thermography. One center preferred LSCI to thermography for acquiring images, and 1 center preferred LSCI to thermography for analyzing images. Conversely, the number of centers preferring standard thermography over LSCI was 3 for acquiring images, and 4 for analyzing images. The remaining centers showed no preference.

**Models including room temperature.** When included as a covariate, room temperature was not associated with any of the summary measures, as measured by either LSCI or thermography. Additionally, the ICCs were not affected by the inclusion of room temperature in the analysis. This does not mean that a regulated room temperature is not important, but that small changes in temperature are acceptable (see Supplementary Table 3, available on the Arthritis & Rheumatology web site at http://onlinelibrary.wiley.com/doi/10.1002/art.40457/abstract).

**Edge effects.** When moving from the thumb to the little finger on imaging, all of the trends in the AUC, MAX, and GRAD were in the opposite direction for the 2 modalities, with a decrease in these values when assessed by LSCI and an increase in these values when assessed by thermography (see Supplementary Table 4, available on the Arthritis & Rheumatology web site at http://onlinelibrary.wiley.com/doi/10.1002/art.40457/abstract) Estimates of the DDD were positive with both techniques, but this was attenuated on images obtained by LSCI. This is consistent with the notion of an edge effect artificially producing lower values for the little fingers when LSCI is used for imaging. The cause of the edge effect was attributed to the distribution of light over the imaging area, due to LSCI being used at the upper limit of the suggested imaging distance in order to fit both hands into the imaging area. These data indicate that care must be taken to understand the variations over the field of view, so that these can be accounted for; decreasing the field of view would minimize this effect in future studies.

## Discussion

To date, LSCI techniques and thermography have been insufficiently studied as outcome measures in clinical trials. Those studies in which they have been included show very little consistency in terms of protocol design 36, 37, 38, choice of dynamic challenge, and extracted outcome measures, making it difficult to compare results between studies or establish a standard protocol. The main finding of our study is that the reliability of both LSCI and thermography (the AUC and MAX) were sufficiently high for use as study outcome measures. The reliability of the MAX_T_ was slightly superior to the MAX_L_. Other than this, there were no substantive differences in reliability between the 2 techniques.

The AUC_M_ and DDD_M_ showed adequate reliability for use as outcome measures. Moreover, there was strong correlation between mobile thermography and standard thermography data. The technique of mobile thermography was added at a late stage in this project (since it had only just come on the market). Our reason for including it was primarily for feasibility assessment. While it is clear that further work is required to validate mobile thermography, the performance in the present study is highly encouraging, because, as a low‐cost tool, it could potentially be readily available for widespread use among rheumatologists.

Although it was not our primary objective, we examined differences between observers. Systematic differences between observers at different centers would not be particularly problematic for a multicenter randomized controlled trial (RCT), provided that the randomization would be stratified by center. We note that this should be the default for any multicenter trial, since differences between centers may otherwise bias the estimated treatment effect. This is particularly true in small populations, since simple randomization is less likely to produce balance within centers. Standardized training would reduce measurement variation across centers, and centralized extraction and analysis of LSCI data, conducted in a blinded manner, might also minimize variation by removing multiobserver differences in an RCT setting. Given the small sample size at each center, we are unable to determine whether truly systematic differences were observed. Ideally, a study to assess interobserver reliability would involve participants having images analyzed by all observers.

Convergence between the techniques was shown to be very high for the AUC and MAX (particularly for the AUC). This finding provides evidence that the same underlying construct is being measured when using these summaries of response. Convergence appeared to be weaker (although still moderate) for the DDD. Convergence was weakest for the GRAD, which may be a reflection of the lag between reperfusion and rewarming, whereby tissue reperfusion (measured using LSCI) is translated into skin rewarming (measured using thermography), during the 2 minutes immediately following cold challenge.

Since there is no gold standard to which we may compare either imaging technique, and we are comparing 2 techniques that measure perfusion by very different methods (skin temperature and a measure of red blood cell concentration and speed by light), it is possible to measure convergence between these techniques for validity 25. It would be unlikely for these 2 techniques to converge if both of them were poor outcome measures, since they would both have to be deficient in distinct but very specific ways, so as to bring the erroneous observations into alignment. Therefore, we can conclude in this instance that their convergence implies validity.

The OMERACT review of 2003 18 assessed the validity of several noninvasive techniques as possible objective outcome measures, but none was deemed ready for use in clinical trials. These techniques included nailfold capillaroscopy, which is a well‐established diagnostic technique now included in the diagnostic criteria to differentiate primary and secondary RP 34. The microscopy technique allows visualization of cutaneous capillaries at the nailbed and identification of the structural changes characteristic of SSc. However, this is not a substitute for functional measures of flow (although functional flow and oxygenation have recently been reported). Plethysmography allows the change in vascular volume to be measured (i.e., detection of a pulse) in combination with cold challenge. The technique can measure full fields in the same way as LSCI, but remains unvalidated. There was no relationship between the summary measures and the RCS on the day of the study visits, for either LSCI or thermography.

Patient‐centered outcome measures are crucial for evaluating the effectiveness (rather than just the efficacy) of treatments. However, patient‐centered outcomes often comprise more “noise” compared to more objective measures of response, and therefore necessitate larger sample sizes to ensure adequate power in clinical trials. For small populations, there is therefore a tension between direct relevance to patients and feasibility of conducting a trial. One solution may be to power studies on the basis of objective measures, such as those considered herein, and to additionally (and consistently) report patient‐centered outcomes to facilitate an eventual meta‐analysis. Another solution might be to seek confirmatory evidence for the vasodilatory potential of candidate interventions, using objective measures, before proceeding to larger, phase III clinical trials.

The relationship between 2 measures is limited by the reliability of each 39. Although the relative stability of the RCS between baseline and follow‐up has been observed in clinical trials/studies 38, 40, there has been little work formally assessing its intraindividual reliability.

With regard to feasibility of the techniques, it has been noted that LSCI is sensitive to movement, vibrations, and lighting, indicating the importance of environmental conditions during the imaging. For mobile thermography, present limitations in feasibility include the battery life (LSCI is mains operated but standard thermography is powered by long‐life batteries), a fixed focusing distance, and lack of analysis for video images, as well as mounting difficulties; however, if the correlation between mobile and standard thermography can be replicated in future studies, these limitations may be acceptable in light of the lower cost and ambulatory (convenient) nature of the technique. When comparing the feasibility of LSCI to the feasibility of thermography, it should be noted that most centers were familiar with thermography but not with LSCI, and therefore this may have influenced the assessment of feasibility.

One limitation of the study was that we did not recruit the planned number of participants, due to a seventh center not participating as planned. However, the study was designed to be robust to under‐recruitment. Although the 95% CIs for our estimates were wider than they would have been had the target been met, we were still able to demonstrate sufficient reliability and convergent validity of the AUC and MAX to observe differences that would indicate that the performance of DDD was weaker, and to show that the performance of GRAD was relatively poor.

In conclusion, our design was relatively pragmatic, with the aim of establishing the performance of the different techniques as they would be employed in a multicenter clinical trial. Our study successfully established a working group of tertiary care centers for SSc, and together, the group developed a consensus calibration and cold challenge protocol. The summary measures of AUC and MAX both displayed good reliability and strong convergent validity. There was a possible advantage of thermography in relation to the reliability of MAX, although this was not definitive. We found evidence of edge effects when using LSCI, although our summary measures appeared to be quite robust to these in relation to reliability, perhaps suggesting that these effects were fairly consistent (details available upon request from the corresponding author).

The results of this study also confirm that small variations in room temperature are acceptable during the imaging, and that, subject to further validation, mobile phone cameras may be a suitable, affordable, and highly portable alternative to more expensive standard imaging equipment (although mobile phones are battery operated and with less functionality [at present] than larger thermal cameras). The mobile phone data obtained in this study will facilitate the design of future validation studies assessing mobile thermography–derived outcome measures. Although the design precluded formal assessment of interobserver reliability, there was a suggestion of systematic differences between the central observer and observers at some of the centers, highlighting the importance of image analysis training and potentially a role for centralized or automated image analysis. For multicenter RCTs, we would also recommend that, where possible or appropriate, randomization be stratified by center to balance any center‐specific effects and prevent bias.

In summary, LSCI and thermography should now be incorporated as secondary outcome measures in upcoming treatment efficacy trials. This will allow an assessment of responsiveness to treatment as well as longitudinal validity. The present study leads us to recommend the summary measures of AUC and MAX, measured using either thermography or LSCI (but especially using thermography), as suitable outcome measures for RCTs in patients with SSc‐related RP.

## Author Contributions

All authors were involved in drafting the article or revising it critically for important intellectual content, and all authors approved the final version to be published. Dr. Murray had full access to all of the data in the study and takes responsibility for the integrity of the data and the accuracy of the data analysis.

### Study conception and design

Wilkinson, Leggett, Marjanovic, Moore, Allen, Anderson, Britton, Buch, Del Galdo, Denton, Dinsdale, Griffiths, Hall, Howell, MacDonald, McHugh, Manning, Pauling, Roberts, Shipley, Herrick, Murray.

### Acquisition of data

Wilkinson, Leggett, Marjanovic, Moore, Allen, Anderson, Britton, Buch, Del Galdo, Denton, Dinsdale, Griffiths, Hall, Howell, MacDonald, McHugh, Manning, Pauling, Roberts, Shipley, Herrick, Murray.

### Analysis and interpretation of data

Wilkinson, Moore, Allen, Anderson, Britton, Buch, Del Galdo, Denton, Dinsdale, Griffiths, Hall, Howell, MacDonald, McHugh, Manning, Pauling, Roberts, Shipley, Herrick, Murray.
