# Supplementary material for: A Multicenter Study of the Validity and Reliability of Responses to Hand Cold Challenge as Measured by Laser Speckle Contrast Imaging and Thermography: Outcome Measures for Systemic Sclerosis–Related Raynaud's Phenomenon
Source: Arthritis Rheumatol. 2018 Apr 23;70(6):903–11. doi: 10.1002/art.40457 (PMC6001804; doi:10.1002/art.40457)
Supplement: Supplementary file 1 [file ART-70-903-s001.docx]

Supplementary Table 1. Inclusion and exclusion criteria.

| Inclusion criteria | Exclusion criteria |
| --- | --- |
| Signed informed consent. | Primary RP or secondary RP due to etiology other than SSc. |
| RP defined as a history of digital cold sensitivity associated with color changes (cyanosis and pallor). SSc as diagnosed by an experienced rheumatologist and fulfilling either the American College of Rheumatology criteria for SSc or the criteria for early disease as defined by LeRoy and Medsger et al . | Inability to undergo LSCI or thermography due to active digital ulcers or inability to extend fingers sufficiently or any other skin features that would lead to. |
| Male and female patients aged ≥ 18 years at screening. | Any significant organ involvement or concomitant condition which, in the opinion of the investigator, would make in unwise for the patient to participate in the study. |
| Stable vasoactive medication: doses stable for at least 1 month prior to Visit 1 and until Visit 2. | Any disorder limiting the ability to provide informed consent or to comply with study requirements. |
|  | Diabetes mellitus which affects the microcirculation. |
|  | Females who were breast feeding or pregnant. |
|  | Smokers; the use of nicotine patches was not allowed as these cause vasoconstriction. |
|  | Change of vasoactive medication within 1 month prior to Visit 1 or planned change of medication between the study visit dates. |
|  | Treatment with prostacyclin (epoprostenol) or prostacyclin analogs (i.e., iloprost, treprostinil) within 1 month prior to Visit 1. |
|  | Local treatment (digits) with botulinum toxin type A within 1 month prior to Visit 1. |
|  | Topical administration of nitrates within 1 week prior to Visit 1. |
|  | Treatment with vasoconstrictive drugs (e.g., ergot derivatives, triptans) within 1 week prior to Visit 1. |
|  | Surgical sympathectomy of digit within 3 months prior to Visit 1. |

Supplementary Table 2. Summary measures of responses to cold challenge comparison of data taken by central and center-specific observers for LCSI and standard thermography.

| Central observer vs center-specific observer | | | | | | | | |
| --- | --- | --- | --- | --- | --- | --- | --- | --- |
| Center | DDD | | Log(AUC ) | | Log(MAX) | | GRAD | |
|  | LSCI (arb PU) | Standard therm. (^o^C) | LSCI (arb PU*time) | Standard therm. (^o^C*time) | LSCI (arb PU) | Standard therm. (^o^C) | LSCI (arb PU/time) | Standard therm. (^o^C/time) |
| Center 2 | 9.0 (-30.0 to 47.8) | 0.02 (-0.92 to  0.96) | 0.09 (-0.35 to 0.53) | 0.02 (-0.07 to 0.10) | 0.10 (-0.19 to 0.39) | 0.00(-0.09 to 0.08) | -3.36 (-16.2 to 9.43) | -0.08(-0.37 to 0.22) |
| Center3 | 48.6 (17.3 to 79.9) | 0.18 (-0.46 to 0.82) | 0.25 (-0.08 to 0.58) | 0.00 (-0.06 to 0.07) | 0.22 (-0.04 to 0.49) | 0.01(-0.05to 0.08) | 1.29(-5.39 to 7.98) | -0.07(-0.46 to 0.31) |
| Center 4 | -15.7 (48.0 to 63.7) | 0.29(-0.76 to 1.34) | -0.15(-0.56 to 0.25) | 0.00(-0.07to 0.06) | -0.12(-0.47 to 0.23) | 0.00(-0.07 to 0.07) | -0.62(-6.93 to 5.69) | 0.05(-0.12 to 0.23) |
| Center 5 | -12.7(-51.6 to 26.2) | 0.11(-0.84 to 1.05) | -0.03(-0.33 to 0.28) | 0.00(-0.06 to 0.07) | -0.01(-0.25 to 0.23) | 0.00(-0.07to 0.08) | -2.56(-17.9 to 12.8) | -0.06(-0.36 to 0.24) |
| Center 6 | -5.5(-21.6 to 10.5) | 0.06(-0.60 to 0.73) | -0.03(-0.23 to 0.18) | 0.01(-0.06 to 0.07) | -0.02(-0.18 to 0.15) | 0.01(-0.06 to 0.08) | -0.32(-5.07 to 4.43) | -0.11(-0.48 to 0.26) |

For comparison of data between observers: Differences in paired means (95% CI), calculated as central minus center-specific. Measurements taken by a central blinded observer were compared to the corresponding measurements taken at each center. The data over both visits for each patient were averaged and the resulting averages between the central observer and the center-specific observer compared by plotting the data and calculating the difference and 95% CI for the paired means. Center 1 is excluded from the analysis as there were multiple observers at this site. The exploratory nature of these supplementary analyses should be emphasized.

Supplementary Table 3. Room temperature data for each center

| Center | One | Two | Three | Four | Five | Six |
| --- | --- | --- | --- | --- | --- | --- |
| Room temperature (^o^C) | 21.9  21.6 to 22.3  20.4 to 22.9 | 23.6  23.5 to 23.7  23.0 to 24.0 | 25.3  24.4 to 24.1  22.6 to 25.6 | 23.9  23.6 to 24.1  22.6 to 25.6 | 23.2  23.0 to 23.5  21.3 to 24.5 | 23.3  22.3 to 24.0  20.2 to 26.9 |

Summary of temperatures per patient visit (data taken over 45 minutes) at each of six centers. Median, interquartile range, range.

.

Supplementary Table 4. Post hoc analysis of the edge effect observed for LSCI

| Summary Measure | LSCI | Thermography |
| --- | --- | --- |
| Log(AUC) | -0.17(0.02) | 0.07 (0.01) |
| DDD | -0.21 (0.02) | -0.01 (0.02) |
| Max | -0.19 (0.02) | 0.07 (0.01) |
| Gradient | -0.07 (0.02) | 0.04 (0.01) |

Estimated trends from index to little fingers, standard error (SEs) from linear mixed models. Units are standard deviations. Positive (negative) trends correspond to increases (decreases) moving across the fingers, with larger values indicating a stronger trend.

Rows show summary measures: Mean (SD); Distal dorsal difference (DDD), area under the reperfusion or rewarming curve (AUC), maximum perfusion or temperature (MAX) and the gradient over the first 2 minutes (GRAD) have been averaged over 8 digits.


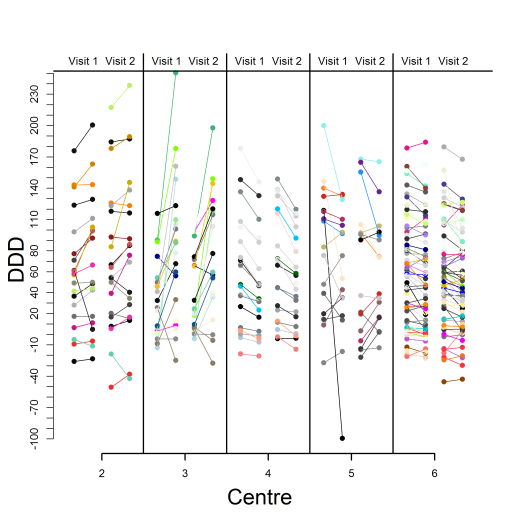

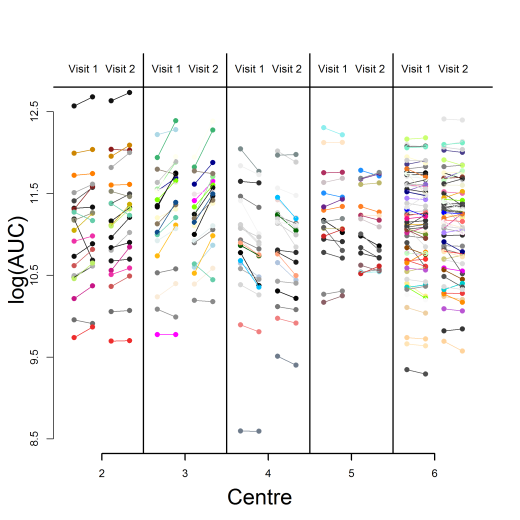

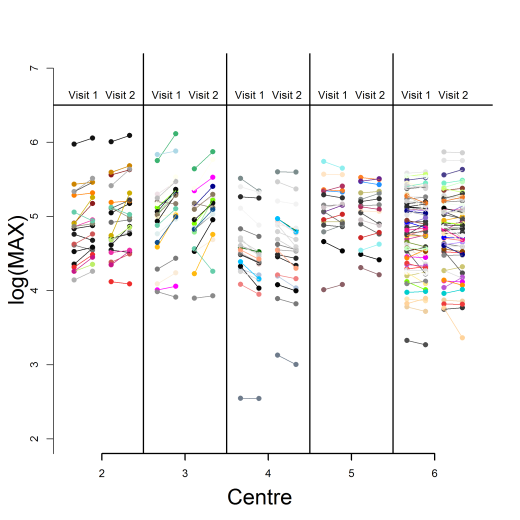

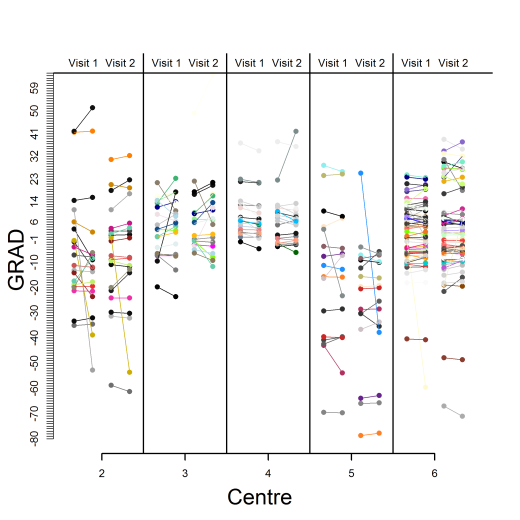


a)


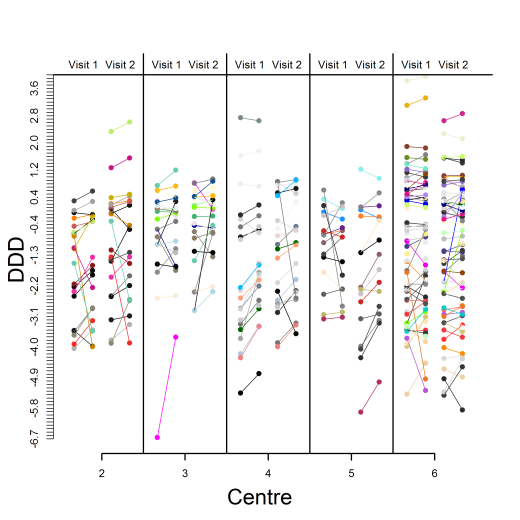

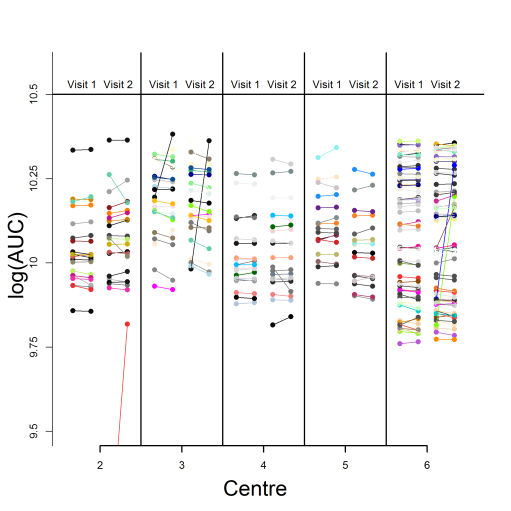

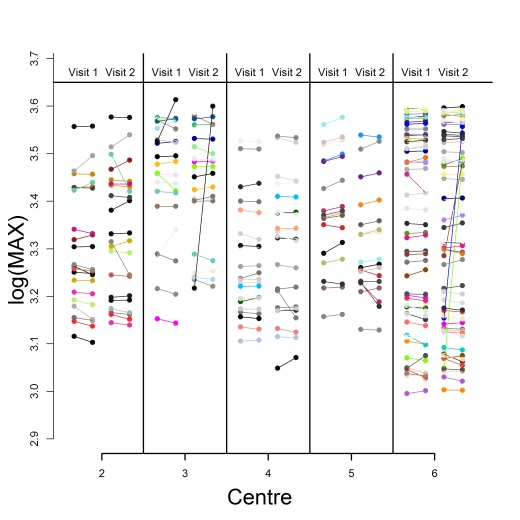

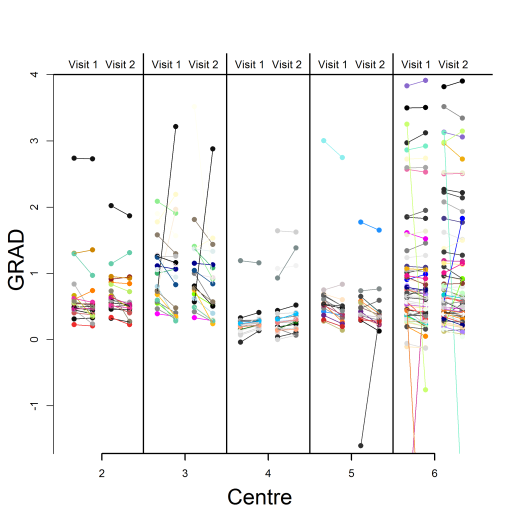


Supplementary Figure 1. Ladder plots showing patient-level summary measures of response measured at each measurement site (first point in each linked pair of points) and again by a universal rater (second point in each linked pair of points). Measurements are shown for each study visit and for a) LSCI and b) thermography.

b)
