# Supplementary material for: A Multicenter Study of the Validity and Reliability of Responses to Hand Cold Challenge as Measured by Laser Speckle Contrast Imaging and Thermography: Outcome Measures for Systemic Sclerosis–Related Raynaud's Phenomenon
Source: Arthritis Rheumatol. 2018 Apr 23;70(6):903–11. doi: 10.1002/art.40457 (PMC6001804; doi:10.1002/art.40457)
Supplement: Supplementary file 2 [file ART-70-903-s002.docx]

**Supplementary data:**

**Expansion of statistical analysis of the data**

*Justification of sample size:* The sample size was selected both to estimate reliability precisely and to demonstrate substantial levels of reliability for thermography in the event that this reflected the actual performance of the technique. A reliability estimate for LSCI was not available, although Murray et al. [22] estimated the reliability of laser Doppler to be 0.92. If LSCI were similar, we would obtain a lower 95% confidence limit of 0.89, again demonstrating strong reliability. In the event that the ICCs of the techniques were considerably lower than expected, a sample size of 180 would still allow estimation of the ICCs to a good degree of precision. For instance, if the ICC was as low as 0.65, then 180 patients would allow a 95% confidence interval of overall width 0.17 to be calculated. The interval would then be 0.56 to 0.73. The inference of the study would then be that reliability of the measurement was ‘moderate’ to ‘substantial’. The target sample size was quite robust to substantial under recruitment. For example, we calculated that, on the basis of the figures presented above, a sample size of 120 would result in 95% CIs for thermography and LSCI of overall width 0.13 and 0.06 respectively, which would still represent a good level of precision.

*Reliability of the techniques:* A fixed effect corresponding to each centre in the study and a patient-specific random intercept were included in the linear mixed effects models. In order to calculate 95% confidence intervals (CIs) for the ICCs a nonparametric bootstrap procedure was performed. In order to compare the reliability of summary measures obtained using LSCI compared to thermography, the difference in ICCs (with bootstrapped 95% CIs) were calculated. AUC and MAX were log transformed due to skewed data prior to modelling. Data from Mobile phone thermography were collected differently and therefore did not lend themselves to calculating all of the summary measures calculated using LSCI and standard thermography. The mean value and the mean DDD were calculated across eight fingers for each patient for each visit (the former a summary measure approximating AUC), and their reliability across visits was computed.

*Reliability between observers:* Measurements taken by a central blinded observer were compared to the corresponding measurements taken at each centre. It is not be possible to calculate a valid inter-observer ICC from this data, which would require at least some of the participants to be measured by all of the observers. Without this, the patient-by-observer interaction is not identified [30]. One centre was excluded from this analysis as several observers analysed the images. The exploratory nature of these supplementary analyses should be emphasized.

*Mathematical representation of the model: T*aking one summary measure at a time, for patient $i$ we have one measurement and one replicate obtained using LSCI ($x_{1i}$ and $x_{2i}$) and one using thermography ($y_{1i}$ and $y_{2i}$).

For $j=1,2$ and $i=1,\ldots,n$ we model

$$x_{ji}=\alpha_{0}+ \alpha_{1}centre_{i}+u_{i}+\delta_{ji}$$

$$y_{ji}= \beta_{0}+ \beta_{1}centre_{i}+v_{i}+ \epsilon_{ji}$$

$\delta_{ji}\sim N(0, \sigma_{\delta}^{2})$ *,* $\epsilon_{ji}\sim N(0, \sigma_{\epsilon}^{2})$ and $\left[ \begin{matrix} u_{i} \\ v_{i} \end{matrix} \right]\sim MNV\left( \left[ \begin{matrix} 0 \\ 0 \end{matrix} \right], \left[ \begin{matrix} \omega_{u}^{2} & \rho\omega_{u}\omega_{v} \\ \rho\omega_{u}\omega_{v} & \omega_{v}^{2} \end{matrix} \right] \right)$

The parameter $\rho$ represents the latent correlation between the two techniques, having separated out the variability due to measurement error. This would be 1 if the techniques were measuring the same underlying construct, and provides evidence of the construct validity of the techniques.
